# A review of open source ventilators for COVID-19 and future pandemics

**DOI:** 10.12688/f1000research.22942.2

**Published:** 2020-04-30

**Authors:** Joshua M. Pearce

**Affiliations:** 1 Department of Materials Science & Engineering and Department of Electrical & Computer Engineering, Michigan Technological University, Houghton, MI, 49931, USA; 2 Équipe de Recherche sur les Processus Innovatifs (ERPI), Université de Lorraine, Nancy, France; 3 School of Electrical Engineering, Aalto University, Helsinki, Finland

**Keywords:** ventilator, pandemic, ventilation, influenza pandemic, open source, open hardware, COVID-19, medical hardware

## Abstract

Coronavirus Disease 2019 (COVID-19) threatens to overwhelm our medical infrastructure at the regional level causing spikes in mortality rates because of shortages of critical equipment, like ventilators. Fortunately, with the recent development and widespread deployment of small-scale manufacturing technologies like RepRap-class 3-D printers and open source microcontrollers, mass distributed manufacturing of ventilators has the potential to overcome medical supply shortages. In this study, after providing a background on ventilators, the academic literature is reviewed to find the existing and already openly-published, vetted designs for ventilators systems. These articles are analyzed to determine if the designs are open source both in spirit (license) as well as practical details (e.g. possessing accessible design source files, bill of materials, assembly instructions, wiring diagrams, firmware and software as well as operation and calibration instructions). Next, the existing Internet and gray literature are reviewed for open source ventilator projects and designs. The results of this review found that the tested and peer-reviewed systems lacked complete documentation and the open systems that were documented were either at the very early stages of design (sometimes without even a prototype) and were essentially only basically tested (if at all). With the considerably larger motivation of an ongoing pandemic, it is assumed these projects will garner greater attention and resources to make significant progress to reach a functional and easily-replicated system. There is a large amount of future work needed to move open source ventilators up to the level considered scientific-grade equipment, and even further work needed to reach medical-grade hardware. Future work is needed to achieve the potential of this approach by developing policies, updating regulations, and securing funding mechanisms for the development and testing of open source ventilators for both the current COVID19 pandemic as well as for future pandemics and for everyday use in low-resource settings.

## Introduction

Coronavirus disease 2019 (COVID-19), caused by a novel coronavirus (SARS-CoV-2), is in part so dangerous because it threatens to overwhelm our medical infrastructure at the regional level, causing spikes in mortality rates ^1– 4^. Within the medical infrastructure, there are critical technologies that are generally available, but simply do not exist in a high enough density to handle the excessive volume of patients associated with pandemics ^5^. Thus, people die unnecessarily throughout the world because of a combination of COVID-19 infections and the lack of access to some of these technologies ^6^. Ventilators are an example of technologies that are currently in critical short supply ^7, 8^. Mechanical ventilators are essential for treating both influenza and COVID-19 patients in severe acute respiratory failure ^9, 10^. Past studies have shown that intensive care units (ICUs) will not have sufficient resources to treat all patients requiring ventilator support during a massive pandemic ^11– 13^, and ethically challenging triage ^14, 15^ would need to be used to decrease mortality over first-come first-served basis for ventilator allocation among patients. Some work has shown promise for using a single ventilator to support multiple patients during a disaster surge ^16– 18^. In addition, it has already been shown that 3-D printed manifolds can assist with rapidly deploying this solution and there are open source designs ^19^. This is not necessarily straightforward ^20^. Although some countries, like the United States, have stockpiles of ventilators ^21^, there is consensus that there is not enough supply for serious pandemics ^22– 25^ and that rationing would be needed ^26^. The current medical system relies exclusively on specialized, proprietary, mass-manufactured ventilators from a small selection of suppliers. This supply model clearly fails when there is a sudden surge in demand for a relatively low-volume specialty product such as ventilators in a pandemic as analyzed here. The vast majority of medical equipment is heavily patented by a few specialty medical firms that sell small volumes because during ‘normal’ times, a medium-sized hospital only needs a handful. These firms have historically aggressively protected their intellectual monopolies ^27, 28^ to the detriment of human lives. In addition, non-practicing entities continue to attempt to actively prevent medical treatments from being deployed, even during the current COVID-19 pandemic ^29^. Putting aside the absurdity of patenting and then obstructing others from using obvious inventions in normal times ^30– 32^, in the wake of a pandemic where millions of lives are at stake, it is intuitively obvious that this type of greed is no longer acceptable.

Fortunately, with the recent development and widespread deployment of open source small-scale manufacturing technologies ^33, 34^, there is now another way – mass distributed manufacturing ^35– 38^. In this new model, designs are developed and then shared with open source licenses freely on the Internet so that others can simply download and replicate the design on their own equipment, even at the household scale ^39^. There has been tremendous and ongoing success of open source scientific hardware proliferation ^40– 45^, where lower-cost and superior-functioning custom equipment as compared to proprietary scientific tools ^46– 49^. Based on such scientific hardware results, there appears to be a significant opportunity to apply open source design principles ^50^ and mass-scale collaborative distributed manufacturing technologies to make medical equipment ^51– 54^. In the current situation, this would at least partially overcome medical supply shortages ^55– 60^ in general, and specifically for ventilators ^61– 63^.

Of these enabling technologies, the most advanced is the fused filament fabrication (FFF)-class of desktop 3-D printers that have spawned from the self- replicating rapid prototyper (RepRap) project ^64– 66^. With the distributed manufacturing model, designs are downloaded even in remote areas and are manufactured on demand as needed ^67^ from readily available (and possibly recycled ^68– 80^) materials. These 3-D printers are, in general, not particularly fast when making products, but with tens of thousands of 3-D printers already strategically deployed all over the world ^81^, they have the capacity to fabricate an incredibly diverse and large range of products (growing exponentially) ^82^, which have already been shared with open source design licenses. Here, the potential will be analyzed for hardware that can be as-much-as-possible digitally manufactured using accessible low-cost fabrication tools like RepRap-class 3-D printers and then readily constructed from widely accessible materials and simple tools (e.g. DIY hardware store sourced along with Arduino-class microcontrollers). RepRap technology in particular is stressed because designs in that ecosystem are already frequently shared, enabling true distributed manufacturing by making use of manufacturing equipment near the point of use. There are, however, many other means of open hardware-based digital distributed manufacturing approaches including CNC mills, laser cutters, engravers, and etchers and other digitally controlled fabrication tools. As pointed out by Mohammed ^83^ many of these tools would overcome limitations of 3-D printing (e.g. speed of replication for flat parts are more easily cut from stock with a CNC tool using subtractive manufacturing than 3-D printing based additive manufacturing).

In this study, after providing a background on ventilators, the academic literature will be reviewed to find the existing and already openly published vetted designs for ventilator systems. These articles will be analyzed to determine if the designs are open source both in spirit (license) ^84^ as well as required practical details. To be open source a ventilator project needs to include:

1) the design source files (e.g. computer aided design or CAD), which are needed to iterate on the design mechanically;
2) as well as production files (e.g. STL files which are used by 3-D printers to make mechanical components);
3) printed circuit board (PCB) layouts and other electronics design files to allow production as well as design evolution of the electronics;
4) bill of materials (BOM), which is needed to allow reviewers to evaluate the components employed as well as more easily find alternatives;
5) list of tools required, which are needed to determine if a device can be fabricated in a specific facility;
6) wiring diagrams, which are used to assemble the device with electronics;
7) firmware and software, which are needed to run the actual device;
8) instructions for the assembly, so makers can fabricate the device when the parts are made or acquired;
9) instructions for calibration as in many cases ventilator designs demand fine tuning to achieve adequate performance;
10) instructions for operation, so the end users can use and maintain the device.

Next, the existing Internet and gray literature will be reviewed for open source ventilator projects and designs. Lastly, as this is a rapidly evolving area, future work will be described to enable wide-spread mass distributed manufacturing of open source ventilators to fight against the current COVID19 pandemic as well as for future pandemics and to provide the devices to low-resource regions of the world that are underserved even in normal times.

## Analysis of literature

Oxygen therapy coupled with mechanical ventilation is meant to support patients so that an adequate oxygen saturation (>88%) in arterial blood is maintained ^85^. The mechanical repository cycle has four parts: 1) inspiration, where the exhalation valve of the ventilator is closed and the ventilator uses pressured air to cause gas to flow into the lungs; 2) cycling, where changeover from inspiration to expiration occurs; 3) expiration, where the main ventilatory flow is interrupted and the exhalation valve opened to allow gas to escape from the lungs, and 4) triggering, where the changeover from expiration to inspiration occurs. According to Andreoli *et al*. ^85^, mechanical ventilators are classified on what factor terminates inspiratory flow, as follows: 1) pressure-cycled ventilators terminate flow when preset pressures are reached in airways; 2) volume-cycled ventilators provide a set volume of gas to the patient over a range of pressures (but a maximum pressure is set to avoid damage to the patient’s lungs during delivery of the set tidal volume); 3) time-cycled ventilators set tidal volume by setting the inspiratory time and flow rate; and 4) flow cycled ventilators, where the inspiratory flow is terminated when the inspiratory flow rate drops below a specific level. The most common commercial modes of mechanical ventilation both provide a specified number of breaths per minute (BPM) and are 1) synchronized intermittent mandatory ventilation (SIMV) where patients can take additional breaths over the set rate and 2) assist control (AC) that uses triggering so that if the patient makes an effort to breathe, it helps them, and if not, it maintains the set rate. These modes can be used alone or in concert with 1) continuous positive airway pressure (CPAP), which uses a high-pressure reservoir and constant flow of gas that exceeds the patient’s needs; 2) positive end-expiratory pressure (PEEP), which increases the residual reserve capacity and allows for many alveoli and small airways to remain open that would otherwise close off; or 3) pressure support ventilation (PSV), which adjusts the pressure on the fly as the patient breathes to maintain a preset inspiratory pressure. For those designing open source ventilators using any of those modes and methods, there is a good base of established literature to draw upon. The classic background is available in Hess, *et al*.’s 1996. *Essentials of mechanical ventilation* ^86^, Tobin’s 2010 *Principles and practice of mechanical ventilation* ^87^, and Owens’ 2018. *The Ventilator Book* ^88^. In addition, Chapter 4 in the openly accessibly book *Equipment in Anaesthesia and Critical Care: A complete guide for the FRCA,* provides a good starting point to help makers understand existing designs and terminology for ventilators ^89^. Texts are available for the use of a ventilator for the standard of care of patients with acute respiratory distress syndrome (ARDS) ^90^, ventilator management for the NIH ^91^, and the practical use of oxygen for patients ^92^. A 2017 state-of-the-art review of mechanical ventilation is presented by Pham *et al*. ^93^ It provides basic schematic diagrams for all of the main classes of commercialized ventilators and reviews their pros and cons.

There exists some confusion on the meaning of the term ‘open source’, which must be clarified to understand how the ventilator designs are evaluated in this review. Ventilators are hardware and thus to be an ‘open source ventilator’, a device must meet the principle and definition provided by the Open Source Hardware Association (OSHWA), specifically:

“Open source hardware is hardware whose design is made publicly available so that anyone can study, modify, distribute, make, and sell the design or hardware based on that design. The hardware’s source, the design from which it is made, is available in the preferred format for making modifications to it. Ideally, open source hardware uses readily-available components and materials, standard processes, open infrastructure, unrestricted content, and open-source design tools to maximize the ability of individuals to make and use hardware. Open source hardware gives people the freedom to control their technology while sharing knowledge and encouraging commerce through the open exchange of designs.”

**Thus, a ventilator (or any other hardware) is not ‘open’ unless it both provides all of the source (as detailed above) to replicate it as well as shares it with a license that protect others’ freedoms to make or use it.** There are some flawed uses of this term from two types of designers. The first type consists of designers claiming they have open source projects before they have shared the code. This is the most rampant in the current ventilator design community with many pretty renderings and high-production value videos with nothing of technical value behind them (i.e. there is no source to replicate the machine available). Most of these designers may have good intentions but the source code may never materialize. Perhaps the most highly publicized case with a good ending was of Medtronic, a large commercial ventilator company, which first announced an open ventilator project on 3-29-2020, but did not release the CAD, BOM, software, etc. to actually fabricate it. Medtronic has now released these documents under a permissive license for their Puritan Bennett 560 ventilator, which already has been commercialized ($10,000 and first introduced 10 years ago) and received FDA approval. Although these design files have been accessed over 90,000 times, this system is designed for mass manufacturing and will likely only be manufactured in that context. All ventilators made from the designs must be labeled with a warning noting that it was built in response to COVID-19, and is only to be used to address this pandemic. Thus, it should be stressed that thisa permissive license is not an open source license. The license only covers addressing the current global coronavirus pandemic, and its term ends either when the World Health Organization’s official Public Health Emergency of International Concern (PHEIC) is declared over, or on October 1, 2024, whichever comes first.

The second type of designers who misuse the term open source, have shared their code, call it ‘open’ but do not actually provide open hardware licenses or they specifically restrict the freedom of others from using it. This confusion is observed throughout the community working on COVID-19-related designs. An example of this confusion is with the ‘make the masks’ website that hosts a 3-D printable mask. They state: “These designs have provisional patents in place, and are intended for this goodwill campaign during the course of COVID-19. If you choose to pursue injection molding, it must be a not-for-profit venture that operates at cost to serve your local community. No license agreements will be awarded to for-profit ventures working to manufacture and distribute this product.” Specifically, in their FAQ it states“…we would like to stress the fact that these files have provisional patents and are for **open source** use only.” This is not what open source means and there is no ‘fair use’ provision for patents as there is with copyright ^94^. In addition, although the STL files are available for replication one must email them for the CAD. This Montana Mask/Billings project is thus not open source by the OSHWA definition. Although it is unquestionably doing some good for the global community because some of the files have been released for distributed manufacturing, it is clearly restricting the end use. The masks take over 3 hours to print on standard 3-D printer and demand for such personal protective equipment (PPE) in some communities outstrips their local supply of 3-D printers. If manufacturers wanted to injection mold them at scale and sell them for a profit while increasing their accessibility and helping people, they are explicitly denied the freedom to do so.

The application of both of these fundamental misunderstandings of what open hardware are have been termed ‘open washing’ or ‘fauxpen’ ^95^. There has thus been a call for open source hardware standardization of practices ^96^ in a way that legally prevents such misuse of the term.

### Existing peer-reviewed literature

The peer-reviewed literature itself is currently limited, but there has been some research on low-cost ventilation, even if the source is not available. First, a field portable ventilator system for domestic and military emergency medical response has been conceptually designed, but does not include enough information to construct it (e.g. the software was written in assembly language and not shared) ^97^. This article does contain design considerations that may be useful for open source designers.

A new, compact and low-cost mask respirator concept has been developed and prototyped successfully ^98^. The blower unit was able to provide adequate ventilation to the test lungs. In addition, the integrated sensor for airway pressure was able to detect airway occlusion and leakages. It is a relatively low-power device and could be operated wirelessly with batteries. It provides a cross-sectional view of the blower unit and some details, but again, not enough to be considered full open hardware or to be easily replicated. It should be noted, however, that many of the components are within RepRap-class 3-D printing capabilities.

In addition, research has been undertaken on a pre-stage public access ventilator (PAV) ^99^. The PAV is made up of several low-cost technologies including a self-designed turbine and a range of sensors for differential pressure, flow, F _i_O _2_, F _i_CO _2_ and three-axis acceleration measurements. The PAV was tested under three conditions to show that it was adequate for an automatic emergency system: 1) pressure-controlled ventilation (PCV), 2) PCV with controlled leakage and 3) PCV with simulated airway occlusion. The PAV was tested for and showed effective ventilation for tidal volume, breathing frequency and inspiratory pressure. Similarly, there has been a proposal to replace artificial manual breathing unit (AMBU) bags with electric blowers to act as emergency ventilators ^100^.

In contrast, another approach is to build a low-cost ventilator utilizing an AMBU bag that is not based on constant blower use ^101^. The study by Mukaram Shahid showed the AMBU setup was able to perform all the functions of a conventional commercial ventilator for a far lower cost (<$100US excluding labor). The automated AMBU device was able to adjust the breathing rate and the volume of the air, which is comparable to older ventilators. However, it was also able to regulate the inspiration to expiration ratio and PEEP rate. Shahid’s system comes with two modes: 1) mandatory ventilation (as in older models) and 2) assisted ventilation (as with most current systems). Thus, the medical personnel can choose to use either the built-in triggering mechanism (assist boosted mode), which alters the respiration pattern once it detects a change in air pressure, or set a time interval for the respiration pattern. The article contains pictures, an electric schematic, a control loop diagram, and very basic results. Again, this can be used as starting point, but there is not enough shared to replicate in the open hardware fashion.

Next, a low-cost ($420 prototype) portable mechanical ventilator was designed and prototyped that delivers breaths by compressing a conventional bag-valve mask (BVM) with a pivoting cam-actuated arm pushed by an electric motor ^102^. This eliminates the need for a person pushing on the BVM, which is generally viewed as only a short-term solution. This system uses knobs to determine the tidal volume appropriate to the patient (usually 6–8 mL/kg of ideal body weight), adjustable BPM of 5–30, and inhalation to exhalation time ratio options of 1:2, 1:3 and 1:4 and a minimum respiratory rate ^103^. This design is run with an open source Arduino micro-controller ^104^ and the article provides enough details to be used as a guide for others to build a similar device, but not the full plans, code, etc. needed to qualify as an open source hardware device.

One of the most relevant designs is a pneumatic ventilator specifically designed for pandemics, which has a low oxygen consumption ^105^. In this study by Williams *et al*., they describe and test three simple, pneumatically powered, low oxygen-consumption ventilators. The three designs were tested for different lung compliances (i.e. different ventilator workloads) on the delivered F _i_O _2_ and oxygen consumption. They used a commercial mechanical test lung for these tests (Vent Aid; Michigan Instruments Inc., Grand Rapids, MI, USA). The results of this study support the potential for mass distributed production of a low-cost, gas-powered, volume-controlled ventilator with a low oxygen consumption (anywhere with oxygen at 2–4 bar). The designs could alternatively be operated on hospital compressed air. The single use, self-inflating bellows system prevents cross contamination among patients. In addition, the system possessed one-way and safety overpressure valves, which could be incorporated into other designs. The designs are in part supplied including basic principle schematics, an example BOM, but falls far short of what is expected for a complete open hardware design.

A large multidisciplinary and international team has just published (currently accepted, available in pre typesetting form) in a study on a low-cost, easy-to-build non-invasive pressure support ventilator meant for under-resourced regions ^106^. The design is based upon using off-the-shelf components and is comprised of an open source Arduino Nano for control, high pressure blower and two pressure transducers. It was bench-marked against commercial systems. Their supplementary material also covers the testing with healthy volunteers, but more importantly, has the basic layout of the device, PCB and circuit schematics including source files, a BOM, STLs for the 3-D printable case, description of the algorithm and the Arduino ino file, and a user manual. This device’s source is available and would represent a method to fabricate a ventilator for <$75, which has already been vetted by medical professionals. There are several interesting points about the approach used ^106^. First, Garmendia et al. took the non-invasive medical approach, which is particularly well suited for both low-income countries ^107^ and also perhaps during pandemics where even the wealthiest nation’s medical systems are strained. By focusing on off-the-shelf components their design could be easily replicated. In ‘normal times’, this approach is second only to systems that can be completely digitally fabricated with local resources. In pandemic situations, it exposes why it is important to have many such designs, as the global supply chains have been disrupted ^108– 110^. Normally, in the U.S. to replicate Garmendia *et al*.’s design based on the documentation provided would only be expected to take a few days. With the disruption, numerous makers have been having trouble sourcing supplies in the U.S., and the lowest-cost blower following the Garmendia *et al.* design has an estimated shipping of 8–18 days on 4-28-2020 in the U.S. There are alternatives for providing this function (both suppliers and devices), which is why it is important to have a ‘diversity of solutions’ ^62^ with as many alternative suppliers, components and possibly even digitally manufacturable parts as possible (e.g. there are already several 3-D printable centrifugal blowers developed, which would demand future work for this application). Lastly, this design did not appear to have a license associated with it being a purely medical science publication. Even the Arduino code, which did have an author information for help, did not contain any license. This could hamper rapid deployment in some contexts as not explicitly indicating a license declares an implicit copyright without explaining how others could use the code ^111, 112^. 

There are also completely different approaches to the design of a ventilator, such as the high-frequency oscillatory ventilator ^113^, but only basic design schematics and preliminary testing is provided. Thus, within the peer-reviewed literature, most of the quasi-appropriate ventilator devices use a standard ventilation bag that is cyclically compressed by either an electromechanic or pneumatic setup and controlled by a microcontroller. Fortunately, the most complicated part of these designs is the controls, which is made accessible by the maturation of Arduino-based microcontrollers that can actuate and sense over a wide array of accessible and already-developed technologies (e.g. code libraries are available). It should be noted that most of the low-cost options in the literature used the bag approach, but that modern commercial ventilators are generally not manufactured with bags, bellows or pistons due to performance concerns. These concerns may be overcome by the nature of a pandemic, as well as by replacing low-cost components during failure, but this does indicate failure detection is warranted and certainly preferred in an open source ventilator design.

### Open source ventilator designs shared on the web

There are a number of proprietary commercial low-cost products like the Pumani bubbleCPAP for infants, D-box or One Breath Ventilators (not yet for sale), which could be used to relieve some of the demand for conventional ventilators. Rather than attempting to conduct a market review of such devices, however, because presumably hospitals facing a shortage of ventilators would already consider all commercially-available and regulated/approved systems, this section will investigate the growing body of knowledge to help makers develop open source ventilators as well as the preliminary designs. This section was largely supported by information gathering of the rapidly evolving open source Internet communities such as Project Open Air, which is a group of “ Helpful Engineers” on the platform Just One Giant Lab. They have congregated to help in the COVID-19 pandemic by developing open source solutions and of most relevance to this study, on a project specifically on the development of open source ventilators. Their documentation and information is freely available. Although just starting, as of 17 March 2020, they have over 2,500 registered volunteers and over 9,000 on their Slack team and by the beginning of April numbered over 15,000. In addition to an offset ventilator, in their first round of project proposals, they have prioritized oxygen concentrators and PPE as their top priority projects. In addition, their future work will focus on tube connectors and building a database for local manufacturers able to produce hardware with high score in reviews.

There are other teams There are other teams working on the development of open source ventilators. Their progress is rapid and there appears to be more groups being formed and joining regularly to address needs in their communities. Robert Read *et al.*, have been attempting to stay on top of these in a COVID-19 Ventilator Projects and Resources with FAQs available on GitHub. This resource contains a color-coded spreadsheet of the various projects and scores them on openness, buildability, community support, functional testing, reliability, COVID-19 suitability, and clinical friendliness and then ranks them by their average score. One can argue with the ranking, but the value of the resource is clear and all projects when they have obtained a reasonable level of development should ask to be evaluated. In addition, the spreadsheet has projects broken down into modular components whenever possible including drivers, monitors, flow sensors, display, oxygen blending and valves.

There are many other teams including those organizing around the open source wiki Appropedia for an open source ventilator, the Air Collective in Bulgaria, EndCoronaVirus.org, the Ventilators Collaboration Network, The Pak Innovation Club, the Oxford-based Ventilator Crowd, 1 Million Ventilators, 1,000,000 Ventilators in 100 days, #EngineersAssemble, and the Ventilator Project. YouTube has over 80 ventilator videos compiled in a list by Kramer. Facebook has an Open Source COVID19 Medical Supplies Group. There is a long-going Pandemic Ventilator Project that hosts their designs on *Instructables.* The RepRap community is starting on an open-source oxygen concentrator, which can be used alone or in tandem with an open source ventilator. *Hackaday* has recently called for a medical hackathon to design and deploy an open source ventilator ^114^. Other communities are crowd-sourcing information about COVID-19 medical technologies and developing a *Coronavirus Technology Handbook*. Some resources for makers are appearing as basic specification provided by Botta. In addition, The Center for Safety, Simulation, and Advanced Learning Technologies (CSSALT) at the University of Florida has started an open source ventilator project based on hardware store components on the assumptions that the FDA will waive clearance for the bare-bones design if there is a massive shortage. The CSSALT system is one of the most professionally documented, with full files available for each sub-system and published engineering specifications for the ventilator that could be useful even for open hardware designers using completely different approaches. They are maintaining their documentation on GitHub. Many of the sub-modules, however, have not yet been developed, nor have a team working on them. Other projects are also using GitHub, like Jackson’s Open Respirator project, but are at the very beginning stages of development as of this writing. To assist these efforts the UK government has issued guidelines.

There are several approaches being attempted in the open ventilator community including pumps, pressure regulators, bellows, pneumatic systems, screw compressors, servo gas modules, fans, blowers, fluid based, cuirass (negative pressure/iron lung), and pistons. The most favored by both the academic literature as well as the maker community is just to use manual ventilators – BVMs/AMBU bags. There are many commercial suppliers available and there is very preliminary documentation for open source manual ventilation for the developing world ^115, 116^. Although, in theory, purely manual ventilation could work to provide ventilation for patients over long periods, there is a real concern of both the availability of the needed man-power, as well as the continued exposure of the laborer. In addition, using a bag-valve mask may increase aerosolization of virus, and in general medical staff are not supposed to bag mask before intubation due to that risk. Many of the open source designs rely on this BVMs/AMBU bags approach where one automates the manual squeezing. It only needs an exhaust system and PEEP valve. Students at Rice University have also created an automated bag-valve mask device that fits around a normal BVM using a dual rack and pinion design with a servo motor that continuously operates (open/close) squeezing the bag a specific amount to supply air. Rice provides a full non-peer-reviewed report, that is considerably richer in details than most of the others. It offers their design strategy, a partial BOM, basic testing, the source code as well as a summary of the standards and regulations necessary to go to market. Unfortunately, in their preliminary testing, the servo motor failed after only 11 hours of service and Rice is withholding the full CAD designs and results. To overcome the limitations of both the MIT and Rice designs, a group in Ireland formed and is moving along with full open source documentation of OpenLung on GitLab. The German language DIY-Beatmungsgerät project They are on their fifth iteration as of this writing based on the surrounding low-cost BVM/AMBU bag concept discussed above. Another project building off the MIT design is DIY Ventilators. Finally, the open hardware OxyGEN project is also using automated AMBU approach and although at the preliminary stages their 3-D and MATLAB design files are hosted openly on GitHub. The OxyGEN current system is under production in Spain.

Makers are also considering other types of non-invasive ventilators (NIV) such as those based CPAP (an alternative to PEEP), which is a form of positive airway pressure ventilator that applies mild air pressure on a continuous basis. A 3-D printed CPAP fan has been designed and tested as a blower and the design files (AutoDesk Fusion 360) and STLs are freely available. Another approach is to turn a commercial CPAP machine into a ventilator currently under development on GitHub by Lee. Lee built the system around an Arduino nano and has performed very basic tests to it that show that it provides enough pressure for a ventilator used on COVID-19 patients; however, there is not nearly enough information to recommend it for medical use. In addition, there are bi-level positive airway pressure (BiPAP) machines that are commonly used at home to treat sleep apnea and lung diseases as they decrease the effort of breathing by changing the pressure for inhalation and exhalation. Home-use BiPAPs could be used in place of hospital NIVs, but care would need to be taken because poor interfaces could generate viral aerosols ^117^. Negative pressure ventilation (iron lung) overcomes this problem, helping lung function by pulling from the outside (there has been some development on Appropedia). It provides a full BOM, but insufficient details for replication or complete open source documentation. 

A unique design currently under development is the ARMEE Ventilator based off of painstakingly recreated Army design ^118^. Without any moving parts the system controls air flow so that its output alternates between two pressure levels. With careful design the pressures, flow rates and cycle times can mimic the output of a mechanical ventilator. This design available on GitHub being licensed a CERN Open Hardware License appears to be particularly amenable to digital fabrication. Other military units have worked on this problem in the past and are doing so currently. An example of another notable design is the AmboVent developed by the Israeli Air Force and now a non-profit. The AmboVent approach is to use a BVM system and aim at mass manufacturing for <$1,000, but their full designs are available on GitHub. Another project meant for mass manufacturing but open source licensed is the Mechanical Ventilator Milano ^119^.

In addition, several makers have developed pandemic ventilators, such as John Strupat, some time ago ^120^, but unfortunately, in addition to the lack of testing, the source does not appear accessible. Another approach is to use a blower, as in the Pandemic Pressure Control Ventilator being developed openly on HackaDay.Io by Frank. Other open source projects are at their beginnings, like the TogRespirator project housed on GitHub developed for a Science Hackday Dublin 2020, DIY and open source respiratory and a project to build an open source ventilator on GoFundMe.

In the review of Internet-reported ventilators, it is somewhat disappointing that many of the most promising designs do not share their source code. Designers that do not share their source making their projects functionally non-replicable. A current representative example would be the Utah-Stanford Ventilator vent4us, which although looking promising and using an innovative a linear actuator-driven pinch valve-based implementation has only indicated they will release their designs in the open source domain, but has not (as of 4-28-2020), despite preliminary evaluation and submission to peer review ^121^. In fact, in many cases, little more than a picture or video are available (e.g. Drexel University’s Dragon Ventilator Project as of 4-28-2020). The newer projects do tend to be following better documentation protocols. Unfortunately, despite the many promising approaches in the maker community, the one problem that the vast majority of the current partial designs have in common is that there is not nearly enough information available about their performance to recommend them for medical use.

### Future work needed

It is clear from this review of the peer-reviewed, gray and open web literature on open source ventilators, that there is considerably more work to do. The tested and peer-reviewed systems lacked complete documentation and the open systems that were documented appropriately were either at the very early stages of design (sometimes without even a prototype) and were essentially only basically tested (and some were not tested at all). With the considerably larger motivation of an ongoing pandemic, it is assumed that these projects will garner more resources and members (as is happening with the Open Air Project) to reach a critical mass to make significant progress to reach a functional and replicable system. Although the motivation of working during a pandemic on a device that may save your life is high, the access to resources, however, is far from optimal. Already, many locations throughout the world are essentially forcing citizens to shelter-in-place, which restricts access to government and university labs, as well as to makerspaces and fab labs. In addition, some areas of the world are suffering from supply disruptions and shipping challenges. This perhaps underscores the importance of developing open source hardware for disasters before the disaster strikes. Future work is needed to develop policies and funding mechanisms for such work as it appears rational to make a small investment in developing and sharing the designs for any critical hardware.

For those planning to work on (or who are already working on) the development of open source ventilators one of the primary challenges is to determine when to share your designs. People are literally dying from lack of ventilators and it is hard for designers not to feel responsible if they are reasonably confident a preliminary device design would possibly prevent those deaths if shared. Many makers follow this belief and often aggressively share their content before there is any evidence that it works. At the same time, well intentioned engineers and designers can have their work mischaracterized and promoted before it is documented by overly aggressive public relations outfits at both companies and universities, which has greatly added to the clutter in this space. On the other hand, as these are medical devices, which literally can mean life and death for a patient, it is reasonable to want to follow the conventional hardware developers’ method: wait to release it until it has been fully tested. In addition, the effort and time it takes to do full documentation correctly may also appear to be lower priority than the making, prototyping and testing of the device itself. However, as Bowman ^122^ points out the “the intent to share a design in the future misses the myriad benefits of open hardware - in terms of scrutiny, feedback, and improvements from the community. It also stifles the development of a community around the design, and there are many cases of promised openness never materialising.” It appears clear that if a project is announced as open source it should include all of the code that is available.

A best practice for open sourcing a design is simply to maintain full documentation of your project as you are working on it in an open platform like GitLab or the Open Science Framework. Designers can start with their designs private, but as parts of the device become ready for ‘show time’ they can click share and everyone can get to the source code immediately. When this is done open announcements can be made to recruit feedback from the community. As ventilators are complex devices made up of mechanical components, electronics, sensors, and firmware/software it is challenging to have a complete device ready (Ideally the systems could reach a maturity in which the subcomponents could be made modular ^123^). These particular devices are also specifically targeted at medical doctors therefore as Farre has pointed out “it is important to make an effort to actively involve health professionals for both design and testing, trying to publish contributions in medical journals” ^124^. The diverse multidisciplinary skill set needed to develop a successful open source ventilator is therefore extremely challenging and explains in part why there is a lack of completed designs. Academic authors can still operate normally with publishing even if it is a hardware related publication. If anything, aggressive sharing before formal peer-review in this way protects precedence and scooping from unethical actors. 

To overcome this challenge of partially completed design release, the method that Appropedia uses for status tagging projects can be borrowed to make it clear what level of development each of the subcomponents is currently at. Appropedia is a website primarily dedicated to developing open source appropriate technology (OSAT) ^125^ for the developing world in a massive collaborative fashion akin to Wikipedia ^126, 127^. Thus, because of the enormous relative investment someone in a developing community must make to fabricate a device a clear designation of the status is provided to readers. The Appropedia Status tags are color code as follows: red for designed, ii) orange for modeled, iii) yellow for prototyped, iv) light green for verified by a specific organization, and v) dark green for deployed listing organizations and numbers of replicants at specific locations in the world. Status tags are placed at the top of a wiki page for a project so that users quickly know what level of risk they must accept to fabricate the project OSAT. For those doing ventilator development on a MediaWiki wiki, the source code from Appropedia for these tag templates can be borrowed immediately as it is under CC-BY-SA, but the concept can be easily adapted to any repository. Following this methodology at the initial writing of this article many of the grey literature devices would be in the red, but as of this update have moved into yellow and presumably will make it to light green and have functional designs verified by someone with an artificial lung shortly (Michigan Tech’s Open Sustainability Technology lab, for example, has offered this for anyone that has developed and shared a fully open source device). The status of the subcomponent and the project as a whole should be clearly visible on the project pages.

It is clear, that open source ventilators would ideally be modular so development can occur in parallel more easily, completely open (by the OSHWA open source hardware definition) with a transparent design and testable with a fully transparent and open validation methodology ideally itself done on vetted open hardware. To assist to democratize these testing steps further there are several efforts to develop an open source artificial lung specifically to test ventilators underway including the VentMon project documented openly on Github and currently at version 0.1 and the Ventilator Inline Sensor Package (VISP) on Hackaday. 

This review article uncovered other limitations to this approach. First, due to potential legal issues challenging an open source ventilator design. It is important that open source ventilators (and other open medical hardware) meet the high-standard levels approved for use in developed countries. The ethics of saving lives, however, require regulation to be more streamlined so that lives are not unnecessarily lost during delays for approval ^62^. Second, because of the general lack of useful technical information in patents (the average Instructable generally has more useful information for constructing a device than a patent despite that being a requirement of obtaining a patent), patents were not included in this review. It should be noted, however, that there are currently over 277 inactive patents in addition to those that have expired covering ventilators. Researchers can obtain this list with direct links to these patents using the Michigan Tech Free Inactive Patent Search ^128^. There may be useful information contained in those documents that could help open source ventilator designers.

Another challenge with this approach is maintaining a proper level of sterility of devices fabricated using distributed means. Specifically, for the FFF-based 3-D printing parts, it has been reported that the prints are sterile at the time of print ^129^. If not kept in a sterile environment, however, they could quickly become biologically contaminated. One approach to deal with this is to use washing or a chemical bath. A relatively complete analysis of the chemical compatibility of commercial 3-D printed plastics is available ^130^. If a specific polymer is needed that cannot be 3-D printed easily, it is possible to make molds in high-temperature plastics, such as polycarbonate, and then use lower temperature plastics to make disposable single use plastic parts ^131^. Similarly, silicone molds can be made from a 3-D printed reverse mold and used in the same way ^132.^

Even when more mature open source ventilator designs are available and can be safely manufactured by a distributed means, another area of critical future work is validation of these designs. In the medical sciences, open source devices like syringe pumps ^133, 134^ are already established ^135– 138^ and have been developed into sophisticated devices ^139– 144^. However, these devices are used in labs in general and not on people continually. For medical professionals to use an open source ventilator, they first must be convinced it will do no harm to them (or others) as well as to the patient. As COVID-19 was reported to spread via droplets, contact and natural aerosols from human-to-human, there has been a concern that high-risk aerosol-producing procedures may put medical personnel at high risk of nosocomial infections, which is a concern for some designs reviewed here ^145^. During the airway management, enhanced droplet/airborne PPE is needed and the study by Zuo *et al*. provides a list of other recommendations to overcome this challenge. There have been some developments in 3-D printing some of these PPE ^146, 147^. Similarly, for designs that could aerosolize the virus, a negative pressure room would be necessary and future work is needed to design an open source approach to creating such rooms. Likewise, the greatest concern for untested open source ventilator designs is that they harm the lungs of the patients; there is significant literature in this area of ventilator-induced lung injury ^148– 155^. There are, however, solutions for preventing this, like controlling the tidal volume ^156, 157^. Thus, the designers of open source ventilators must ensure that their designs have safety features to prevent ventilator-induced lung injury, as well as having basic testing of the prototypes to ensure that the designs themselves are thoroughly vetted. This must be done using yet-to-be-developed transparent process controls and quality assurances as well as tests. There has been some preliminary efforts in this direction for minimal qualifications based on the UK guidelines and the more than 70 COVID-19 Ventilator Validation Tests under development by Public Invention. More work, is needed however, to make freely accessible standards and protocols for testing, quality assurance and use of ventilators. Today such information is behind a complex web of paywalls from standards vendors, equipment suppliers, and copyright holders from publishers, universities and medical vendors and in the worst case are simply proprietary trade secrets. A future review is needed to find pathways to accessible sources of reliable information in each of these areas to accelerate the development of low-cost and free and open source medical hardware of every kind including ventilators

Within the open source scientific equipment community, such procedures are relatively well established and have been working reasonably well through normal peer review of hardware-based articles like those published by *HardwareX*, the *Journal of Open Hardware*, and *PLOS One*. For medical-equipment that could be all that stands between life and death, this vetting is even more important and open calls for papers for a Special Issue on Open-Source COVID19 Medical Hardware are attempting to address this.

However, technical validation may not be enough. Medical hardware used on humans is also more complicated, as any studies involving humans needed to verify its functionality on people, need institutional review board approval and, if in regulated areas like the U.S., such a study would need an Investigational Device Exemption to allow for a non-FDA approved device to be used as part of a study. This is only a temporary approval and the full device would need actual FDA approval for legal deployment unless the laws are changed (or were temporarily suspended during a pandemic). These same regulatory roadblocks are in place in other nations, which has conventional ventilator manufacturers skeptical that even conventional manufacturers of other products (e.g. vacuum cleaner and automobile manufacturers are doing this now in the UK) could switch over to produce ventilators ^158^. Clearly, this process is a problem during a pandemic. Both for the current situation and during potential future situations, there is a need to limit liability on the part of the designers, makers and users of such open source medical hardware ^159^. One approach is for ‘Good Samaritan’ laws to be expanded to protect both the makers and designers of open source medical hardware ^62^. Substantial future work is needed in this area. Finally, it should be pointed out that personnel and training can become limitations to deploying mass medical efforts, even if open source ventilators are available. So, future work is needed to create training materials and translate it into the languages spoken throughout the world as well.

## Conclusions

There is clear technical potential for alleviating ventilator shortages during this and future pandemics using open source ventilator designs that can be rapidly fabricated using distributed manufacturing. The results of this review, however, found that the tested and peer-reviewed ventilator systems lacked complete documentation (with one recent exception) and that the current open systems that were documented were either at the very early stages of design or had undergone only early and rudimentary testing (although this is changing rapidly). With the considerably larger motivation of an ongoing pandemic, it is assumed these projects will garner greater attention and resources to make significant progress to reach a functional and easily replicated open source ventilator system. There is a large amount of technical future work needed to move open source ventilators up to the level considered adequate for scientific-grade equipment and further work still to reach medical-grade hardware. Future work is needed to achieve the potential of this approach not only on the technical side, but also by developing policies, updating regulations and securing funding mechanisms for the development and testing of open source ventilators for both the current COVID19 pandemic, as well as for future pandemics and for everyday use in low-resource settings.

## Data availability

No data are associated with this article.
