# Supplementary material for: A review of open source ventilators for COVID-19 and future pandemics
Source: F1000Res. 2020 Apr 30;9:218. Originally published 2020 Mar 30. [Version 2] doi: 10.12688/f1000research.22942.2 (PMC7195895; doi:10.12688/f1000research.22942.2)
Supplement: Peer review history

# Peer review: Reviewer response for version 2

**DOI:** 10.5256/f1000research.26243.r62939

**Published:** 2020-05-07

**Authors:** Mazher Mohammed

**Affiliations:** 1 School of Design and Creative Arts, Loughborough University, Loughborough, UK

Upon reviewing the comprehensive modifications made by the author, I am deeply satisfied the present manuscript should be accepted for indexing.

Is the review written in accessible language?

Yes

Are all factual statements correct and adequately supported by citations?

Yes

Are the conclusions drawn appropriate in the context of the current research literature?

Yes

Is the topic of the review discussed comprehensively in the context of the current literature?

Yes

Reviewer Expertise:

Medical Devices, Bioengineering, Product Design, Sustainable Technologies and Materials, Open Source Hardware

I confirm that I have read this submission and believe that I have an appropriate level of expertise to confirm that it is of an acceptable scientific standard.

# Peer review: Reviewer response for version 2

**DOI:** 10.5256/f1000research.26243.r62938

**Published:** 2020-05-01

**Authors:** Ramon Farre

**Affiliations:** 1 Unit of Biophysics and Bioengineering, School of Medicine and Health Sciences, University of Barcelona, Barcelona, Spain

No more comments

Is the review written in accessible language?

Yes

Are all factual statements correct and adequately supported by citations?

Yes

Are the conclusions drawn appropriate in the context of the current research literature?

Yes

Is the topic of the review discussed comprehensively in the context of the current literature?

Yes

Reviewer Expertise:

Respiratory mechancs, Respiratory medical devices, Biomedical engineering.

I confirm that I have read this submission and believe that I have an appropriate level of expertise to confirm that it is of an acceptable scientific standard.

# Peer review: Reviewer response for version 1

**DOI:** 10.5256/f1000research.25328.r61802

**Published:** 2020-04-22

**Authors:** Mazher Mohammed

**Affiliations:** 1 School of Design and Creative Arts, Loughborough University, Loughborough, UK

**Introduction**

 The manuscript presents a review of open sources ventilators with particular emphasis towards applications in the COVID-19 pandemic during the early phases of 2020. Given the submission date of the manuscript, which has coincided with the emergence of the COVID-19 pandemic, the review is very topical, in addition to being thought provoking and insightful. COVID-19 disease is caused by severe acute respiratory syndrome coronavirus 2 (SARS-CoV-2), where the presenting symptoms of a patient are predominantly based upon respiratory ailments, requiring ventilation based equipment to treatment the most severe of cases. As the virus infiltrates a greater frequency of the general population, the availability of ventilation based products has rapidly become one of the primary causes for concern not only in developing, but in developed economics. Given the disruption of standard medical device supply chains during a pandemic, a viable alternative to meet the demand for ventilation equipment from healthcare providers is to turn to communities of designers, engineers, industrial specialists and knowledgeable maker enthusiasts to develop easily accessible, low-cost and open source alternatives to traditional devices. The manuscript is therefore a vital piece of documentation to assist researchers in global efforts to create ventilator alternatives. Equally, the manuscript does an exceptional job at highlighting the current state of the art in this area with balanced and considered conclusions throughout. **I would therefore very much recommend this article for indexing, which will be of great interest to the scientific community and those seeking to develop their own open source solutions.**

**Recommendations and Thoughts**

 It is noted that the article is based on the premise of discussing ventilator technology which are deemed open source. As review manuscripts generally attract a wider target audience that scientific bodies of work, it would seem appropriate for the author to define what is meant by open source, for the benefit of unfamiliar readers. Indeed, one may see general scientific publications are open source information by virtue of the information being in the public domain. However, it is clear that the authors perception of this would require a deeper level of ‘disclosure’ of the innovations presented by various research groups to allow for ready duplication and adoption of such systems. This is starkly evident later in the manuscript during discussions of existing literature. Therefore, the distinction of what constitutes open source in this context should be explicitly defined, ideally within the introduction.

 In light of the rapidly evolving nature of the pandemic and the volume of initiatives that are attempting to provide viable, often, open source solutions, there is likely to be some developments that have not been addressed in the review. Generally, the author has done an exemplary job of drawing the readers attention to many of the most topical and noteworthy examples. However, one would imagine as we approach the end of the pandemic period that there is likely to be a wealth of additional technologies which will have surfaced and so perhaps a follow up review may be justified, ideally inviting multiple authors involved in such projects to contribute. It is however noted that certain information within the manuscript has since evolved, specifically relating to the comment in the first paragraph of the introduction, where it is mentioned that *‘…….for a recent example, consider the fact that a manufacturer threatened to sue a maker for 3-D printing life-saving valves in Italy for patent infringement….’* Upon further development of this story, the company had made official statement to clarify that they did not attempt to sue the party which made ventilator valve parts but had primarily withheld designs based upon medical device regulation. Although withholding designs during this particular circumstance when supply chain needs could not be met resulting in potential mortality of patients, there is an argument to disclose such information despite legal implications. However, this is very much different to the notion of the company suing the Italian firm. At the time of writing the article this would not have been known to the author, but given the controversial nature of the comment, I would request the author to reword this sentence to reflect the final outcome of this case study.

 During the introduction when discussing the very many technologies available to the open source community, the discussion preferentially revolves around the use of rep-rap 3D printing, under the notion of digital fabrication technologies which have distributed manufacturing potential. This is a very important point to make by the author as distinctions are made as to why this approach would provide added value within the context of a pandemic. In particular, designs may be shared both at a national and international level using internet based data transfer, while leveraging manufacturing and technical capacity closer to the point of use. Such capacity has long been utilised by the open source community, providing strong resilience in instances when typical supply chains are disrupted, as would be the case during a pandemic. However, this discussion appears to be somewhat incomplete for readers who are unfamiliar with this approach of manufacturing. More specifically, it may be useful for future readers to hear some mention of other digitally driven distributed technologies, such as milling/CNC machining, laser engraving/etching and other digitally controlled tools. Indeed, several of these technologies would serve to reduce or eliminate the perceived limitations of 3D printing both in terms of manufacturable materials and speed of manufacturing. Please could the author include some additional discussion here to contextualise available options to the open source community with respect to digital and distributed manufacturing.

 The author presents an eloquent attempt to discuss both the academic and non-academic ventilation systems, citing many interesting studies and raising most of the key facets of each respective technology. Following from the previous comments regarding the open source nature of a given study, it is highlighted that sadly many academic studies, though presenting some remarkable feats of engineering, simply do not provide sufficient information to allow for other researchers and experts to duplicate a respective ventilation system. This is indeed a shame and highlights perhaps a necessity for researchers to adopt a more open frame work of reporting academic findings and equally for reviewers to encourage and accept such ways of reporting. In light of major global health catastrophes such as pandemics, such openness within the literature may in fact prove advantageous to hasten innovations to tackle the detrimental effects on a given population.

 The discussion on the current open source efforts appears to cover several of the major projects to the awareness of this reviewer, but more crucially provides a good cross section of important developmental aspects to inform the reader. As highlighted previously, given the rapidly evolving nature of the pandemic and the wiliness and passion of researchers and technical experts, new attempts to create ventilation products are arising on a weekly, if not daily basis from groups around the world. Therefore, in this reviewer’s opinion, to cover all such attempts would be impractical but also unnecessary given that the overlap of technical development with existing projects. As with the previous section, it would have been good to present annotated diagrams and a summary chart/table of the systems broken down into the primary attributes that fulfil the requirement for ventilation. In reading this section it was difficult to surmise how these efforts were truly moving towards a functional ventilation device. One of the more noteworthy examples from Rice University, with credible data to back the developments to date was sadly hindered by component failure after only a 11-hour evaluation period, which falls considerably short of a functional ventilator. Clearly, several if not all examples highlighted by the author raise several notes of caution toward open source design, namely the clarity and robustness of evaluation, which lacks the rigour of typical academic scrutiny in addition to the lack of standardisation of components which are suitable for purpose. Arguably, the author to varying degrees’ highlights this by stating the case studies in question are either in the very preliminary stages of investigation, show a lack of relevant performance data or a Bill of Materials (BOM) which enables scrutiny of the components employed. I believe there is a missed opportunity of discussing these elements in greater depth, which is vitally required if open source ventilators are to truly enter mainstream acceptance and use. I would very much welcome further discussion on limitations with appropriate recommendations, which both do not stifle the breadth of design ideas by the community, but also provide substantiative guidance to direct those involved to be mindful of critical milestones and ‘codes of best practise’ during the journey from inception of idea to final working and ‘usable’ ventilator. For example, looking at open source efforts for Personal Protective Equipment (PPE) we see that the 3D Printing community rapidly converged towards the preferential use of PETG polymers for manufacturing, owing to the factors of mechanical stiffness providing a semi flexible yet robust part, and importantly biocompatibility for limited human contact and being food grade to allow ease of decontamination. Equally, strict protocols were developed to minimise contamination of parts during printing, handling and shipping to both reduce the spread of the virus within the supply chain and to follow some element of best practise, similar to constraints set by medical device companies upon their manufacturing procedures. It would be very useful to the open source community if the author would share their thoughts in more explicit detail to provide a template that could be built upon for future efforts, outlining best practise from initial design ideation to working prototype. Arguably, the imperative of this is much greater than with PPE given potential intrusive nature of ventilation systems providing a clear route to internal infection through the lungs.

 One element that appears to be missed in the present review is an overview of current commercial systems and evaluation of their performance characteristics to be both efficacious and crucially to prevent unintended harm to a patient. The author does cite an existing review of commercial ventilators by Pham *et al*, and so it would be unnecessary to conduct a repeat of this work. However, what would be relevant in the present manuscript is a discussion focusing on the regulatory and quality assurance aspects and how these would align and differ in the approach of open source ventilation systems. Indeed, commercial devices must adhere to the very strictest regulatory scrutiny to be classified and used as a medical device, particularly given the invasive nature of their operation to either supplement or take complete control of a patients breathing. As such there are tightly regulated frameworks of ‘fail safes’ to ensure that every component used falls within acceptable usage limits, that construction of such devices follow strict regimes for assembly and minimisation of contamination, that tests are conducted to evaluate the working performance of each device, that there is a robust training and best practise usage protocol, amongst many other safety measures and supply chain demands. Arguably, one of the biggest reservations by the commercial sector regarding open source hardware for medical device technology are the lack of process control, quality assurances and regulations regarding technological development and best practise. It would be valuable for the readers to have some insight into what safety and evaluation procedures that are conducted and how such measures would be replicated in open sources systems in general terms, highlighting key challenges which can be built upon for the future discussion.

 The final sections of the review offer an exceptional summary of the state of art in the field of open source ventilators, highlighting several challenges and opportunities in this space. The author rightly highlights the very early stages open source ventilations systems currently are at and that we should not expect these systems to be working within a clinical setting in the immediate future. Despite this, I for one feel optimistic given the current work that is underway, our ability to access a wealth of digital knowledge, alongside the availability of hardware and manufacturing resources at our disposal.  Arguably, the open source community has never been better equipment to make positive impact on the world during the COVID-19 pandemic. It is also the opinion of the reviewer that there remains many more opportunities for both growth of the open source community and to leverage the expertise synergistically with other academic groups to more rapidly advance our preparedness for emergency situations.

 Reflecting upon the manuscript, I could not help but feel there are differing schools of thought that of the traditionalist and the open source innovator. The traditionalist will operate with robust scientific rigour but will provide limited information scientific manuscripts, be open to patent and potentially restrict free flow of concepts and with respect to medical technology will strictly follow medical regulatory frameworks. The open source innovator is generally driven by an overwhelming sense of openness and transparency in their work, with the belief this will help proliferate and see ideas adopted faster for anyone’s benefit, albeit on some occasions operate with a naivety towards regulatory and best practise aspects. Initial discussions by the author drew distinction between efforts of the academic and non-academic communities, which very loosely are comprised of these two types of innovators, with the exception of a few ‘maverick’ academic groups. However, there was little discussion of strategies as to how these two communities may come together in cooperation and bridge any perceived differences in thinking. Clearly the academic community prides its outcomes based on empirical evidence, the careful scrutiny of data, alongside objective design performance metrics, attributes that the authors clearly outlines as shortfalls of the general open source community. Conversely, the non-academic community provides a wealth of creativity, ingenuity, alongside technical prowess, finding often remarkable and highly efficacious solutions working with limited resources and minimal dependency of specific supply chains. I would therefore strongly welcome the author to add further discussion towards strategies on how both communities, which for the most part work independently, could align agendas to realise opportunities that transcend the sum of the two parts. Indeed, I think it more critical for the open source community, based upon the reflection of the author, to be more engaged in traditional scientific process and to incorporate this into their thinking to hasten product development for evaluation.

 One element that gave me considerable food for thought, was the notion that developing nations have a perceived advantage during global health emergencies due to their more relaxed legal and regulatory frameworks to deploy open source ventilator systems. I am not entirely convinced that this is the case and indeed the author makes several valid arguments to the contrary, mainly that the technology is not significantly mature to function as intended and without consequence. Such suggestions can indeed have several unpleasant connotations from a legal and ethical standpoint and so I would encourage the reviewer to consider an amendment to this comment. Ultimately, I believe it was not the authors intension to imply this given previous discussions, however the context of this point should be framed better.

**Summary**

 Overall, despite the sombre theme of the review, the author has done an admirable job of bringing together all the relevant themes relating to open source ventilation systems. One of biggest take home messages from the review is how much potential exists with the open source community to provide cost effective, robust and timely medical device solutions, which may be far less susceptible to supply chain disruption and leverage a greater capacity for localised fabrication using the distributed manufacturing model. This capacity can only be realised by continued development of existing open source projects, increased dialogue with academic groups to work collaboratively to validate and iteratively improve ventilation system concepts for maximum efficacy. Equally, there is a clear need for regulatory reform which appreciates the evolving circumstances during a global health crisis and could provide an alternative framework to leverage capacity outside of typical medical supply chains to supplement efforts on the ground, as and when appropriate. What this framework should look like is another debate entirely, but this article makes an elegant argument for the debate to be had.

 Despite the infancy of open source ventilation systems, much potential exists and it is an exciting time for developers to continue their efforts towards working solution. It is exciting to see what may be a paradigm shift in how we perceive and operate globally in the medical device sector, particularly in light of the recent issues during the COVID-19 pandemic, which have decimated supply chains, while the shear volume of cases has put a drain on medical resources. The pandemic has already seen the use of open source designs, manufactured in a distributed manner, make impact to supplement shortfalls in PPE equipment. Could the same in time be true for ventilator technologies based on the balance of growing demand and available resources? Only time will tell. I reiterate the relevancy of the article by the author and the manuscript has been a very thought provoking document to read and digest. I would whole heartedly recommend this article for publication and encourage researcher and technologist in the field to draw inspiration from the insightful and thought provoking arguments outlined. I do welcome a time when the lessons we are learning during the pandemic lead to a more caring and equitable world for us all, and it feels from a technological standpoint, that open source innovation will be part of that story.

**Additional minor points for consideration**

- The hyperlink for Ref 82 needs revising as it links to an error page.
- One the first paragraph of page 4 ‘breadth’ has mistakenly been used in place of ‘breath’. Additionally there are some minor grammatical errors toward the end of this same paragraph that need amending, specifically the sentence starting with ‘texts area available for the ……..’
- Generally speaking, it would have been very useful to contextualise much of the discussion with annotated diagrams of several key open source innovations, to give the reader a real feel for the types of devices in development. Indeed, this is typical of many academic based reviews of the scientific and gray literature. Unfortunately, this has not been the case in the present manuscript and I would invite the author to consider such an amendment.

Is the review written in accessible language?

Yes

Are all factual statements correct and adequately supported by citations?

Yes

Are the conclusions drawn appropriate in the context of the current research literature?

Yes

Is the topic of the review discussed comprehensively in the context of the current literature?

Yes

Reviewer Expertise:

Medical Devices,  Bioengineering, Product Design, Sustainable Technologies and Materials, Open Source Hardware

I confirm that I have read this submission and believe that I have an appropriate level of expertise to confirm that it is of an acceptable scientific standard, however I have significant reservations, as outlined above.

# Response

**Published:** 2020-04-28

**Authors:** Joshua M. Pearce

**Affiliations:** Michigan Technological University, USA

Thank you for the rigorous and exceptionally detailed review.

 Following your recommendation, I have added a much more explicit section detailing the meaning of open source as well as provide examples of it being misused.

 I have removed the now incorrect example of the manufacturer suing over reverse engineered valves. Although it may be interesting to note I contacted the individual who reversed engineered them before the initial article was published and I have yet to receive the STL files.

 I share your frustration with the current lack of appropriate sharing. I have included a more detailed review of the existing designs – but as you point out it is impractical to do everything in such a rapidly changing field where websites are being updated hourly or faster. I have declined to develop diagrams - even if I could find appropriately licensed images as a summary because of this rapidly changing nature. Particularly in mechanical designs because those appear to be the most often altered.

 I have expanded considerably what the files are for in open hardware to help better introduce those outside of the open hardware community to the concept as well as make it clearer why they need to be included.

 I have brought in an example from the PPE community.

 In addition, following your recommendation I have attempted to provide some best practices in terms of ‘when to share’ in the discussion. This is meant to bridge the gap between the traditionalist and maker philosophies you discuss.

 After considering your points and those of the other reviewers about the advantages of less developed regulatory systems I have simply removed this entirely.

 I have corrected the minor mistakes you pointed out – thank you for finding them.

 Lastly, I don’t think that a complete review of all the legal hurdles and regulatory framework needed in this technical space can be done here - it needs a completely separate review. We have a major problem here because even some of the standards are not available. When an artificial lung company contacted their customers on my behalf to get a testing protocol they referred me to a long list of ASTM standards which I was not able to acquire through my relatively-well-resourced University library. I purchased the first one and was disappointed to find that it mostly contained references to other standards and a shocking dearth of useful technical information. As there is a conflict of interest between the publishers of such standards and providing all the information in a single location that is freely available for designers to help in a pandemic, this represents one of the largest hurdles. Some good-willed publishers have provided open access to some of their documents during the pandemic. This is a nice start but a better approach is to use public funding to provide open access documentation as the cost and opacity of these standards provide a barrier to open source ventilator development and as I pointed out an unacceptable state during an emergency.

# Peer review: Reviewer response for version 1

**DOI:** 10.5256/f1000research.25328.r61800

**Published:** 2020-04-21

**Authors:** Ramon Farre

**Affiliations:** 1 Unit of Biophysics and Bioengineering, School of Medicine and Health Sciences, University of Barcelona, Barcelona, Spain

This review by Prof. Pierce is an excellent and timely piece of work. I think the author is to be commended for this panoramic perspective on the current state of the art in the open source field of mechanical ventilators for acute respiratory failure. In addition to subscribing to Prof. Bowman's extensive comments, I would particularly like to emphasize how important it is that open access contributions regarding medical devices be made in the context of multidisciplinary cooperation between professionals from different fields. In fact, unlike many other open access development areas, where the proposed devices or products can be used by a wide variety of users, medical devices focus on very specific end users: the doctors responsible for diagnosing and treating the patients. Therefore, any proposal for open access in medical devices must be not only technically sound and cost-effective, but, most importantly, safe and clinically applicable in real-life clinical routine. Consequently, as clearly stated in Professor Pierce's paper, realistically testing new developments is a critical step. Unfortunately, it is not unusual for some new open access contributions for medical applications to be the result of developments by authors drawn exclusively from the technological field, with the result that the proposed devices exhibit applicability limitations that seem obvious to clinicians who are potential users. Therefore, to advance the extension of the open access approach, it is important to make an effort to actively involve health professionals for both design and testing, trying to publish contributions in medical journals, not only in publications focused exclusively on open access. A very recent work, exactly in the field of developing open access mechanical ventilators for resource-poor areas, shows an example of a team of professionals covering the fields of biomedical engineering, respiratory physiology, physiotherapy and respiratory clinical medicine ^1^. This multidisciplinary team has conceived, developed and tested a novel non-invasive pressure support ventilator following the conventional method of evaluating ventilators (bench tests in simulated patients and preclinical evaluation in volunteers) and has published the results in a prestigious journal of pneumology. To end my review of Prof. Pierce's excellent work, I would mention an open ethical issue about the requirements for medical devices to be used in resource-poor areas. It is important that these devices have the same high standard level as those approved for use in developed countries (e.g. CE / FDA marks). But it is also important that until the industry can provide such medical devices at affordable prices for LMICs, patients are not deprived of life-saving therapies. Finding a balance on this ethical-legal issue is difficult but fundamental.

Is the review written in accessible language?

Yes

Are all factual statements correct and adequately supported by citations?

Yes

Are the conclusions drawn appropriate in the context of the current research literature?

Yes

Is the topic of the review discussed comprehensively in the context of the current literature?

Yes

Reviewer Expertise:

Respiratory mechancs, Respiratory medical devices, Biomedical engineering.

I confirm that I have read this submission and believe that I have an appropriate level of expertise to confirm that it is of an acceptable scientific standard.

## References

[1] : Low-cost, easy-to-build non-invasive pressure support ventilator for under-resourced regions: open source hardware description, performance and feasibility testing. *European Respiratory Journal*.2020; 10.1183/13993003.00846-2020 10.1183/13993003.00846-2020 PMC717367232312862

# Response

**Published:** 2020-04-28

**Authors:** Joshua M. Pearce

**Affiliations:** Michigan Technological University, USA

Thank you for the careful and constructive review.  

 I have included your well-reasoned point about the need for multi-disciplinary collaboration, the need to involve medical personnel, and to aim to publish in the medical literature.

 In addition, I have included a deep review of Garmendia et al. design. A sincere thanks for pointing this out as well as for the work itself.

 Thank you for the point about ethics – to account for this I have added several points about this in the discussion.

# Peer review: Reviewer response for version 1

**DOI:** 10.5256/f1000research.25328.r61799

**Published:** 2020-04-02

**Authors:** Richard Bowman

**Affiliations:** 1 Department of Physics, University of Bath, Bath, UK

This is a really good, thorough review of open source ventilators, including both a good technical background, a review of existing peer reviewed projects, and a summary of current efforts. I would thoroughly recommend it to anyone considering joining or using an open source ventilator project, as it's particularly good at pointing to some relevant literature that describes the requirements and principles of operation. The conclusion of the article is that we're not there yet - most of the published designs are not sufficiently complete to be easily replicated, while most of the current open projects are not rigorously tested.

 The review of peer reviewed articles is interesting and does a good job of rating the different solutions in terms of openness; it is disappointing that these articles don't generally give sufficient information to reproduce the ventilator, but also unsurprising. This lends a great deal of weight to the current move towards more openness in science, where protocols, data, and schematics can be shared in data archives along with papers - but of course that's rarely done retrospectively.

 The review of "internet and gray literature" seems objective and reasonable to me, and while such a review cannot possibly stay exhaustive given the frequency with which such projects are appearing, it does seem to cover many of the projects I've heard of. More important than an exhaustive list, however, is the discussion of the common issues to most of the DIY projects - the need for careful testing, quality control, and proper authorisation. Most discussions have focused only on technical validation - but as the author rightly points out, this is not the only way medical devices must be assessed.  At least as pressing as the technical challenge is the difficulty of getting new suppliers and new devices through a quality assurance process that gives medical professionals the confidence that they can safely use said devices.

 Openness is an important, and often surprisingly contentious, issue. Of the projects that are discussed, only relatively few make available complete designs for their solution. This is particularly surprising in the case of some projects from high-profile institutions that have already been widely reported in the media as "open" while not yet having released any designs. The commonly-accepted practice in open software is that complete designs, including source code and documentation, are made available to the public, and that a project is not considered open until this happens. Similar norms are being established for open hardware projects, supported by organisations such as OSHWA and GOSH.

 Given the safety-critical nature of a ventilator, it's reasonable to be reluctant to release untested designs out of a desire to be responsible. Given the time-critical situation, sharing documentation and designs may also be considered lower priority than product development. However, the intent to share a design in the future misses the myriad benefits of open hardware - in terms of scrutiny, feedback, and improvements from the community. It also stifles the development of a community around the design, and there are many cases of promised openness never materialising. My own view is that projects ought not to claim openness until their designs are publicly available under an appropriate license, but there are definitely valid ethical and practical concerns here, and I would welcome an open debate on the best way forward. 

 The one statement in the article that I'm slightly troubled by is the suggestion that developing countries may be at an advantage due to their less robust regulatory systems. Firstly, while it is true that many countries in the Global South do have less formal economies, their regulations are often very tightly aligned with those in richer nations - for example, the Tanzanian medical device regulations closely mirror those used in the EU. If different standards are adhered to, it may be because the regulations are not implemented fully, rather than because the government has intentionally applied lower standards. Also, the better-resourced regulatory bodies in rich nations are more able to accelerate the process of approval if needed; it is not clear to me that a medical device would clear the bureaucratic hurdles and achieve approval any faster in a developing country, indeed the process can be much slower. It is also a very thorny ethical issue to trial medical interventions in the Global South that would not pass ethical scrutiny in richer nations, particularly as the interventions are often being proposed by people from said richer nations. I don't think the author is suggesting this, but I do feel it's a point worth highlighting. While there is often an argument made that low quality medical supplies may be better than nothing, it is also reasonable to expect that developers of technology shouldn't do anything to citizens of Low and Middle-Income Countries (LMICs) that they wouldn't do to patients in their own nation. Indeed, most ethical review panels in the UK apply exactly this criterion.

 The challenge of creating a safety-critical medical device that can be produced in a distributed manner is significant, and I think the article reflects this. I could not agree more with the statement that "technical validation may not be enough" and would probably go further, to say that technical validation alone is not sufficient to ensure patient safety. While many open ventilator projects now exist and have gathered impressive numbers of volunteers, there remains a significant global challenge to enable such projects to be regulated appropriately, either in the current crisis or longer-term. The existing system of medical device regulation is slow, expensive, and conservative; while this conservatism has its roots in the entirely reasonable desire to prevent harm to patients, the way the system is implemented makes it extremely difficult to certify a medical device without the resources of a large company. Reform of these regulatory systems could enable a more agile approach to the design and manufacture of safety-critical components, but a satisfactory supply chain will also require significantly more quality management than is present in a typical "maker space" run by volunteers, hobbyists, or even experienced engineers.  Questions around training and liability are also of paramount importance; while litigation against volunteers acting in good faith seems unduly harsh, there must be accountability in the supply chain of medical devices. Otherwise, we push responsibility onto the clinical staff using uncertified equipment, which adds a crippling burden to front-line staff who are already working at the limit of their capacity.

 Overall, I think it's right to keep an optimistic tone, while acknowledging the obvious difficulties associated with the current challenge. It's likely that, while there are many 3D printers available around the world, formal structures that do not yet exist will be needed to enable them to be fully employed to solve supply issues in this and future crises. Whether or not it is possible to make use of community designed and built ventilators in the coming months, I look forward to a world where critical supplies can be designed and produced openly for the common good. If we take the opportunity to put LMICs on a more equitable footing with respect to richer nations, the future may be more inclusive, as well as more resilient.

Is the review written in accessible language?

Yes

Are all factual statements correct and adequately supported by citations?

Yes

Are the conclusions drawn appropriate in the context of the current research literature?

Yes

Is the topic of the review discussed comprehensively in the context of the current literature?

Yes

Reviewer Expertise:

Automated microscopy and open source hardware

I confirm that I have read this submission and believe that I have an appropriate level of expertise to confirm that it is of an acceptable scientific standard.

# Response

**Published:** 2020-04-28

**Authors:** Joshua M. Pearce

**Affiliations:** Michigan Technological University, USA

Thank you for the thorough review and kind comments. I agree with you that the state of full open source disclosure in the general literature is disappointing and share your hope that the current move towards open science continues to accelerate.

 In this revised version I attempted to pull the grey literature review up to date, but as you point out that is a daily battle and I have cited the work by Read et al., trying to do that on Github.   

                                                                                 

 I share your surprise in the wave of what can only be described as “open washing” where high profile organizations announce their development of an open source ventilator, which upon closer inspection is not open and/or not all of the critical files have been shared. To address this, I have greatly expanded the introductory information and definition of openness as well as provided a few examples.

 I have also offered additional thoughts on “when to share” in the Future Work Needed Section.

 After considering your points and those of the other reviewers about the advantages of less developed regulatory systems I have simply removed this entirely.
